# Clinical and Molecular Epidemiology of Staphylococcal Toxic Shock Syndrome in the United Kingdom 

**DOI:** 10.3201/eid2402.170606

**Published:** 2018-02

**Authors:** Hema Sharma, Debra Smith, Claire E. Turner, Laurence Game, Bruno Pichon, Russell Hope, Robert Hill, Angela Kearns, Shiranee Sriskandan

**Affiliations:** Imperial College, London, UK (H. Sharma, D. Smith, C.E. Turner, S. Sriskandan);; MRC London Institute of Medical Sciences, London (L. Game);; Public Health England, London (B. Pichon, R. Hope, R. Hill, A. Kearns)

**Keywords:** staphylococcal toxic shock syndrome, bacteria, MRSA and other staphylococci, *Staphylococcus aureus*, TSST-1, CC30, *ccpA*, United Kingdom, antimicrobial resistance

## Abstract

Staphylococcal toxic shock syndrome (TSS) was originally described in menstruating women and linked to TSS toxin 1 (TSST-1)–producing *Staphylococcus aureus*. Using UK national surveillance data, we ascertained clinical, molecular and superantigenic characteristics of TSS cases. Average annual TSS incidence was 0.07/100,000 population. Patients with nonmenstrual TSS were younger than those with menstrual TSS but had the same mortality rate. Children <16 years of age accounted for 39% of TSS cases, most caused by burns and skin and soft tissue infections. Nonmenstrual TSS is now more common than menstrual TSS in the UK, although both types are strongly associated with the *tst*+ clonal complex (CC) 30 methicillin-sensitive *S. aureus* lineage, which accounted for 49.4% of all TSS and produced more TSST-1 and superantigen bioactivity than did *tst+* CC30 methicillin-resistant *S. aureus* strains. Better understanding of this MSSA lineage and infections in children could focus interventions to prevent TSS in the future.

## Abstract 2

Nonmenstrual TSS is now more common, and better understanding will improve prevention interventions.

Staphylococcal toxic shock syndrome (TSS) is a life-threatening illness characterized by fever, rash, desquamation, organ dysfunction, and shock. In 1980, the use of highly absorbent tampons in the United States triggered an outbreak of menstrual TSS (mTSS) in young women, and TSS incidence peaked at 13.7/100,000 population (*1*). Changes in tampon manufacture and advice regarding tampon use helped halt the epidemic. TSS is a notifiable illness in the United States; in 2004–2014, average annual incidence varied from 0.03–0.05/100,000 population (*2*). In the United Kingdom and other countries in Europe, staphylococcal TSS is not a notifiable illness, so the clinical, microbiological, and toxigenic features of TSS remain poorly described.

TSS is attributed to staphylococcal superantigens that cause massive T-cell activation and cytokine release (*3*). TSS toxin 1 (TSST-1) is associated with 95% of mTSS cases and 50% of TSS cases caused by nonmenstrual infective foci (nmTSS) (*4*). Although 24 different staphylococcal superantigens have been described, including staphylococcal enterotoxin (SE) and enterotoxin-like superantigens (*5*), SE types A, B, and C are implicated in remaining nmTSS cases (*3**,**6*), despite the lack of data from Europe.

TSST-1 is encoded by the gene *tst*, which is carried on mobile genetic elements (MGE) named staphylococcal pathogenicity islands (SaPIs) that lie within the *S. aureus* chromosome. SaPIs are linked to specific *S. aureus* genetic families, known as lineages (*7*). Within human *S. aureus* strains, *tst* is carried on SaPI1, SaPI2, and SaP68111 (*8**,**9*). Known regulators of *tst* include the *S. aureus* accessory gene regulator operon (agr) via the effector molecule RNAIII (*10*), the staphylococcal respiratory response regulator AB (SrrAB) (*10*), a glucose catabolite repressor CcpA (*11*), the staphylococcal accessory regulator A, σ^B^ (*12*) and the SaeRS 2-component system (*13*).

mTSS strains are reported to belong to a single *S. aureus* lineage (*14**,**15*) corresponding to multilocus sequence type–clonal complex (MLST-CC) 30, a lineage prevalent in the United Kingdom (*16*). Staphylococcal methicillin resistance is mediated by *mecA* or *mecC* genes within the mobile genetic element staphylococcal cassette chromosome *mec* (SCC*mec*), of which there are 12 types (*17**,**18*). Methicillin-sensitive *S. aureus* (MSSA) and methicillin-resistant *S. aureus* (MRSA) strains that are members of CC30 carry *tst* on SaPI2 (*19**,**20*).

In this study, we aimed to characterize the clinical and molecular epidemiology of TSS in England, Wales, and Northern Ireland. We further determined superantigen production by dominant *S. aureus* strain types.

## Methods

### Case Identification

Public Health England (PHE) requests the referral of all TSS-associated isolates to the national reference laboratory for characterization, including toxin gene profiling. We identified clinician-diagnosed staphylococcal TSS cases from a database of referred *S. aureus* isolates from England, Wales, and Northern Ireland during January 2008–December 2012 using the search term “toxic shock syndrome.” Clinical and demographic data from the accompanying isolate referral form (Technical Appendix) that had been recorded contemporaneously were scrutinized for accuracy by a clinician (H.S.) before inclusion in the study.

We classified TSS cases in patients <16 years of age as pediatric. We classified cases in female patients 12–60 years of age as mTSS if the infection was associated with menstruation or positive vaginal culture for *S. aureus*. We classified the remaining cases as nmTSS. All cases had an associated *S. aureus* isolate.

The average annual incidence of TSS was calculated as cases per 100,000 population using Office for National Statistics UK population estimates (http://www.ons.gov.uk/ons/datasets-and-tables/index.html) and was based on data from 2009 and later (due to changes in reporting practice from November 2008 prompted by national guidance on toxin-producing *S. aureus*). We used total population for the United Kingdom excluding Scotland as the denominator for all TSS and nmTSS cases; the total female population 12–60 years of age as the denominator for mTSS cases, reflecting the age range of this group; and the number of children <16 years of age as the denominator for pediatric cases. We included data from 2008–2012 in all other analyses.

### Molecular Characterization of Isolates

We made MLST-CC assignments on the basis of sequencing the staphylococcal protein A (*spa*) gene repeat region (*21*) and referencing *spa* server (http://spa.ridom.de/mlst.shtml) and MLST (http://saureus.mlst.net) databases. We performed SCC*mec* detection, typing, and toxin gene profiling (*sea-e*, *seg-j*, *tst*, and *pvl* only) by multiplex PCR (*22**,**23*).

### Antimicrobial Susceptibility Testing

For isolates from 2008–2011 (n = 148; Technical Appendix Table 1), we determined antimicrobial MICs by agar dilution (*24*) and interpreted them in accordance with European Committee on Antimicrobial Susceptibility Testing guidelines (http://www.eucast.org). We did not determine antimicrobial susceptibilities for isolates from 2012.

### TSST-1 Production

Based on molecular epidemiologic findings, we assessed TSST-1 production in all *tst*-positive CC30 MSSA isolates from the TSS cohort (n = 81), including TSS isolates associated with bacteremia, skin and soft tissue infections (SSTI), and deep infections. We also assessed TSST-1 production in randomly selected *tst*-positive CC30 MRSA isolates from non-TSS patients (n = 39, including carriage, bacteremia, and SSTI isolates) that had been submitted to the reference laboratory during the study period (Technical Appendix Table 1). We quantified TSST-1 in cell-free broth-culture supernatants by Western blot by comparison with purified TSST-1 protein standards (Technical Appendix).

### T-Cell Proliferation

We obtained normal-donor peripheral blood mononuclear cells (PBMC) from an approved subcollection of the Imperial College NHS Trust Tissue Bank (ICHTB reference R12023) from anonymized consenting healthy donors. We incubated PBMC (1 × 10^6^ cells/mL) with cell-free RPMI bacterial supernatants (1:1,000 dilution) prepared from *tst*-positive CC30 MSSA isolates from the TSS cohort (n = 77; 4 of the isolates did not grow in RPMI) and the randomly selected *tst*-positive CC30 MRSA isolates (n = 39) that were investigated for TSST-1 production. We cultured the PBMC in RPMI medium (Invitrogen, Hemel Hempstead, UK) supplemented with 10% fetal calf serum at 37°C for 48 h in triplicate (*25*). We measured proliferation after incorporating 1.0 μCi/well of [^3^H] thymidine and allowing an additional 16 h incubation.

### DNA Sequencing and Analysis

We extracted whole genomic DNA from randomly selected *tst*-positive CC30 MSSA isolates from the TSS cohort (n = 4) and *tst*-positive CC30 MRSA isolates (n = 5) (Technical Appendix Table 1) (*26*). We prepared libraries using the Nextera-XT DNA Sample Prep Kit (Illumina, Cambridge, UK) and subjected them to MiSeq sequencing (Illumina), generating 150 bp reads. We deposited data in the GenBank short read archive (accession no. SRP082305). We mapped reads to MLST-CC matched reference genomes MRSA252 (GenBank accession no. NC_002952.2 (*27*) or MN8 (accession no. NZ_CM000952) using SMALT (http://www.sanger.ac.uk/resources/software/smalt/) and determined single-nucleotide polymorphisms (SNPs) by SAMtools and bcftools (*28*). We performed de novo assemblies using Velvet (https://www.ebi.ac.uk/~zerbino/velvet/) and annotated them using Prokka (http://www.vicbioinformatics.com/software.prokka.shtml). We used Artemis (http://www.sanger.ac.uk/science/tools/artemis) to visualize the mapping of sequence reads to the reference strain and manually confirm all polymorphisms. For targeted *ccpA* sequencing, we amplified and sequenced DNA using forward primer 1: 5′- CACAGTGTCGCGTGTTGTTA-3′ and reverse primer 1: 5′- TAAGCGCATCCCTACTGCAC-3′.

### Statistical Analysis

We analyzed data with GraphPad Prism 6.0 (GraphPad Software, La Jolla, California, USA). We tested categorical variables using Fisher exact test or χ^2^ test. We summarized nonparametric data by medians and interquartile ranges (IQR) and compared 2 groups by Mann-Whitney U test. We summarized parametric data by means and SDs and analyzed 2 groups by t-test (2-tailed); we considered p<0.05 significant.

## Results

### Incidence of TSS

During January 2008–December 2012, a total of 195 TSS case isolates were referred to PHE. We excluded 15 cases from the study (duplicate isolates from the same case, 4 cases; isolates submitted for quality control testing, 2 cases; isolates from cases incorrectly recorded as TSS, 9 cases), leaving 180 microbiologically confirmed TSS cases with isolates. Because of missing clinical data, we were unable to classify 3 isolates as mTSS or nmTSS and could not ascertain the sex of 1 patient with nmTSS. 

**Figure 1:**
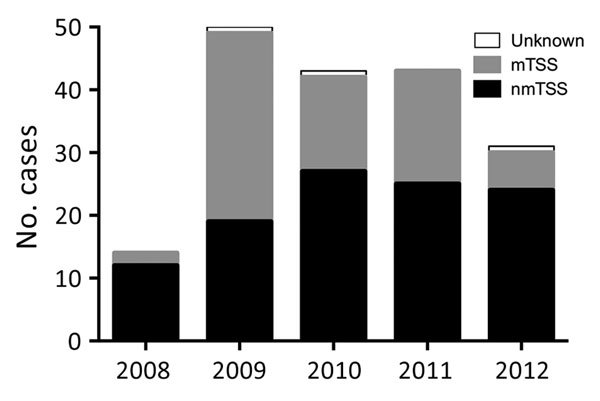
Staphylococcal TSS cases, England, Wales, and Northern Ireland, 2008–2012. The chart depicts the number of cases per year of total, menstrual, and nonmenstrual TSS cases reported to Public Health England. National guidance on toxin-producing *Staphylococcus aureus* disease affected reporting practice from November 2008. mTSS, menstrual TSS; nmTSS, nonmenstrual TSS; TSS, toxic shock syndrome.

 We considered the apparent rise in cases during 2008–2009 an artifact of increased clinical awareness of severe toxigenic *S. aureus* disease from late 2008, prompted by national guidance on toxin-producing *S. aureus* (Figure 1). Beginning in 2009, mTSS referrals declined annually, whereas nmTSS cases remained stable. By 2012, cases of nmTSS outnumbered mTSS. Overall, most cases were nonmenstrual (107, 59.4%). Average annual incidence per 100,000 population was 0.07 (95% CI 0.05–0.10) for all cases, 0.09 (95% CI 0.06–0.14) for menstrual cases, and 0.04 (95% CI 0.02–0.06) for nonmenstrual cases.

### Clinical Characteristics of TSS Patients

Despite an overall preponderance of female case-patients, we found no gender difference among nmTSS cases (Table 1). The median age of the cohort was 19 years; patients with nmTSS were younger than those with mTSS (median 15.0 vs. 21.5 years; p = 0.01).

**Table 1:** Clinical characteristics of staphylococcal toxic shock syndrome cases, United Kingdom, 2008–2012*

Characteristics	All patients, n = 180†	Menstrual, n = 70	Nonmenstrual, n = 107	p value
Median age, y (IQR)	19.0 (9.0–38.3)	21.5 (17–35.3)	15.0 (1–43.5)	**0.01‡**
Sex, no. (%)				
F	128 (71.1)	70 (100)	55 (51.4)	**0.0001§**
M	51 (28.3)	0	51 (47.7)	
Unknown	0	0	1 (0.9)	
Deaths, no. (%)	9 (5.0)	4 (5.7)	5 (4.7)	0.74**§**

*Boldface indicates a statistically significant result, p<0.05. IQR, interquartile range.  †3 patient isolates not assigned as menstrual or nonmenstrual due to lack of clinical data ‡Mann-Whitney U test comparing menstrual and nonmenstrual TSS cases. §Fisher exact test comparing menstrual and nonmenstrual TSS cases.

**Figure 2:**
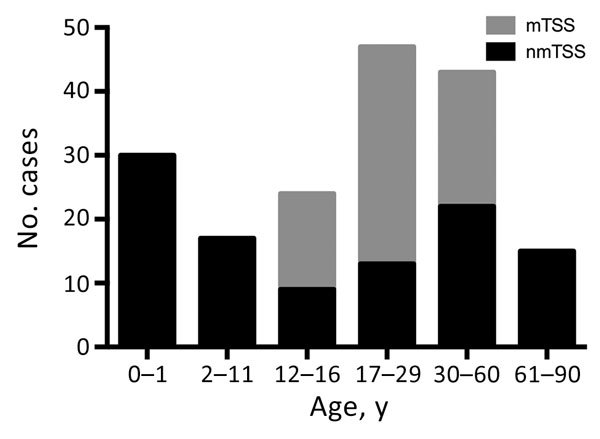
Age distribution of patients with staphylococcal TSS in England, Wales, and Northern Ireland, 2008–2012. mTSS, menstrual TSS; nmTSS, nonmenstrual TSS; TSS, toxic shock syndrome.

Of the TSS cases studied, 39% (71/180) occurred in children <16 years of age; one sixth of all TSS case-patients were <1 year of age (Figure 2). The median age of pediatric TSS case-patients was 4 years, with an average annual incidence of 0.14/100,000 children (95% CI 0.08–022). However, among children <1 year of age, the average annual incidence increased to 0.45/100,000 (95% CI 0.26–0.79). Most pediatric nmTSS cases were related to burns (26.8%, 15/56) or SSTIs (25%, 14/56). 

Five percent of all patients with TSS had died at the time of referral of the isolate. We found no difference in fatality rate between mTSS and nmTSS cases and no association with age (Technical Appendix Table 2). The infective focus in nmTSS cases was SSTI (n = 41), primary bacteremia (n = 15), burns (n = 15), deep abscess (n = 13), respiratory tract (n = 10), bone and joint (n = 4), unknown (n = 6), and other sites (n = 3). We found no association between site of infection and *S. aureus* lineage (Technical Appendix Figure 1).

### Molecular Characteristics of TSS Isolates

**Figure 3:**
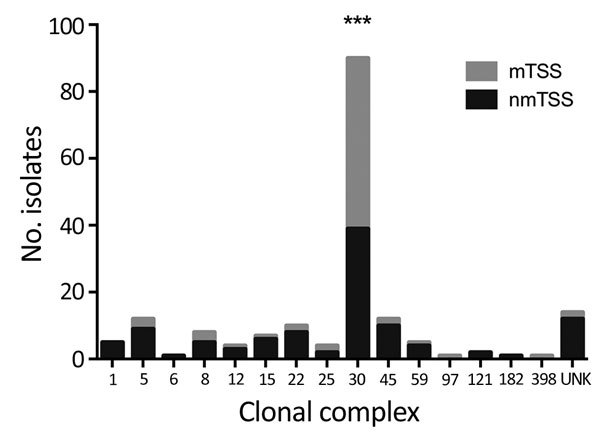
Number of isolates from each *Staphylococcus aureus* clonal complex causing staphylococcal toxic shock syndrome in England, Wales, and Northern Ireland, 2008–2012. ***p<0.0001 by Fisher exact test. mTSS, menstrual TSS; nmTSS, nonmenstrual TSS; TSS, toxic shock syndrome; UNK, unknown (isolates that failed to grow on subculture).

Among 180 TSS *S. aureus* isolates, we identified 88 *spa* types associated with 15 different MLST-CCs (Technical Appendix Table 3). The leading cause of both mTSS and nmTSS was CC30 MSSA, accounting for >50% of infections (Figure 3), although we found a stronger association of CC30 with mTSS than with nmTSS (72.9% vs. 36.4%; p<0.0001; Technical Appendix Table 3). CC30 MSSA was also the leading cause of TSS among pediatric cases (31/71). We identified only 7 MRSA TSS isolates (Technical Appendix Table 4).

TSS isolates carried 3 superantigen genes on average (Technical Appendix Table 5). The most common superantigen gene among both mTSS and nmTSS isolates was *tst* (Table 2; Technical Appendix Figure 2), with the exception of the other 2 prevalent superantigen genes, *seg* and *sei,* that are carried on an enterotoxin gene cluster (*egc*) along with *selm/n/o/u* in most *S. aureus* isolates (*5*). The *tst* gene was associated with mTSS (Table 2) and strongly associated with the CC30 lineage of *S. aureus* (Technical Appendix Table 6). The superantigen gene *sea* combined with *tst* was also linked to mTSS (Table 2), whereas *sea* alone was associated with CC30 (Technical Appendix Tables 5, 6); *sec* was linked to nmTSS (Table 2) and CC45 (Technical Appendix Table 5). Ten nmTSS cases were associated with isolates that lacked any superantigen gene tested; 7 were CC15, highlighting severe disease attributable to this lineage that was unexplained by the presence of major superantigens (Technical Appendix Tables 5, 6).

**Table 2:** Frequency of major superantigen genes among *Staphylococcus aureus* isolates associated with menstrual and nonmenstrual toxic shock syndrome, United Kingdom, 2008–2012*

Superantigen gene†	No. (%) cases			p value§
	Total, n = 180‡	Menstrual, n = 70	Nonmenstrual, n = 107	
*sea* and *tst* combined	54 (30.0)	27 (38.6)	25 (23.4)	**0.04**
*tst* alone	37 (20.5)	23 (32.9)	13 (12.1)	**0.001**
*sea* alone	12 (6.7)	4 (5.7)	8 (7.5)	0.77
*seb* alone	11 (6.1)	3 (4.3)	8 (7.5)	0.53
*sec* alone	14 (7.8)	1 (1.4)	13 (12.1)	**0.01**
*sed* alone	4 (2.2)	0	4 (3.7)	0.15

*Boldface indicates a statistically significant result. †Does not include 48 TSS isolates that did not have *sea, seb, sec, sed,* or *tst* in isolation.  ‡Three additional TSS isolates could not be classified as menstrual or nonmenstrual due to lack of clinical data §By Fisher exact test comparing the percentage carriage of a given superantigen gene among menstrual and nonmenstrual isolates.

### Antimicrobial Susceptibility of TSS Isolates

Most isolates were MSSA (*mecA* negative). The rate of resistance to erythromycin was 9.2%; to ciprofloxacin, 8.5%; to tetracycline, 3.5%; and to teicoplanin, 1.4%. For 7 *mecA-*positive MRSA-TSS isolates, the resistance rate to ciprofloxacin was 57.1%; to erythromycin, 42.6%; and to clindamycin, 14.3%.

As MRSA-related TSS is rarely reported we examined these cases in more detail. All 7 MRSA cases were nonmenstrual, affecting mainly male patients; 3 were associated with SSTIs. The median patient age was 34 (IQR 2.3–64.3) years. Five isolates were identified as CC22-SCC*mec*IV, and 4 carried *sec,* corresponding to the healthcare-associated MRSA clade dominant in the UK, EMRSA-15; MRSA-TSS cases showed a clear association with this lineage (Technical Appendix Table 4). The remaining CC22 isolate carried *tst* and belonged to a MRSA lineage frequently identified in the Middle East. Only 1 MRSA-TSS isolate was CC30-SCC*mec*II, corresponding to the UK HA-MRSA clade EMRSA-16. One isolate was CC6-SCC*mec*II and lacked all superantigen genes tested.

### TSST-1 Production by CC30 *S. aureus*

The strong association of CC30 with TSS was unsurprising because of the presence of *tst.* We measured TSST-1 in broth-culture supernatants from *tst*-positive CC30 MSSA isolates from the TSS cohort and, for comparison, randomly selected clinical *tst*-positive MRSA isolates that belonged to the same lineage (CC30) (*20**,**29*) (Technical Appendix Table 1).

**Figure 4:**
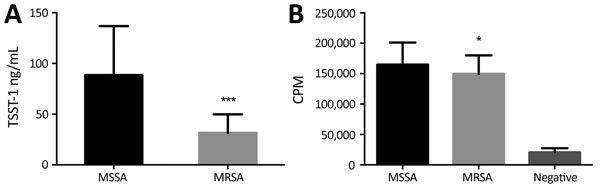
TSST-1 and total mitogen production in vitro by *tst*-positive clonal complex (CC) 30 MSSA and CC30 MRSA strains. A) Mean TSST-1 present in the culture supernatants of *tst*-positive CC30 MSSA (n = 81) and CC30 MRSA (n = 39) isolates measured by immunoblot after overnight culture in brain–heart infusion broth. B) Mean human PBMC proliferative response to culture supernatants of *tst*-positive CC30 MSSA (n = 77) and CC30 MRSA (n = 39) isolates. Negative indicates RPMI tissue culture medium (Invitrogen, Hemel Hempstead, UK) alone. Error bars indicate SDs. *p<0.05; ***p<0.0001 (both by 2-tailed t-test). cpm, counts per minute; MSSA, methicillin-sensitive *S. aureus*; MRSA, methicillin-resistant *S. aureus*; PBMC, peripheral blood mononuclear cells; TSS, toxic shock syndrome; TSST-1, TSS toxin 1.

Of note, 77/81 *tst*-positive CC30 MSSA isolates produced detectable TSST-1, compared with 9/39 *tst*-positive CC30 MRSA isolates. The *tst*-positive CC30 MSSA isolates produced more TSST-1 than did *tst*-positive CC30 MRSA isolates, albeit with marked variability (88.5 ± 48.3 vs. 31.4 ± 18.1 ng/mL,;p<0.0001; Figure 4, panel A). Furthermore, the superantigenic activity of isolates, measured by T-cell proliferation in response to broth-culture supernatants, of *tst*-positive CC30 MSSA strains (164,893 ± 36,191 counts/min) was significantly greater than that of *tst*-positive CC30 MRSA strains (149,653 ± 30,412 counts/min; p = 0.02; Figure 4, panel B).

### *tst*-positive CC30 MRSA and Mutation in *tst* Regulator, CcpA

To ascertain the basis for the observed variability in TSST-1 production among CC30 *S. aureus,* we subjected 4 *tst*-positive CC30 MSSA isolates from the TSS cohort and 5 *tst*-positive CC30 MRSA clinical isolates to whole-genome sequencing. The *tst* gene, promoter, and regulator sequences, including SarA, SrrAB, agr, and σ^B^, were identical among the 9 sequenced strains and reference isolates (MN8/MRSA252).

We detected mutations in TSST-1 regulator SaeRS in 2/4 *tst*-positive CC30 MSSA isolates; a synonymous SNP C481T in SaeR in 1 strain and a nonsynonymous SNP in SaeS in another resulted in a change from asparagine to serine at aa residue 218. Because these strains produced abundant TSST-1 (Technical Appendix Table 1), we did not study these mutations further.

We detected a nonsynonymous SNP in the sequence of regulator *ccpA* in all 5 *tst*-positive CC30 MRSA isolates but not in any *tst*-positive CC30 MSSA isolate. This difference translated into a change from threonine (ACA) to isoleucine (ATA) at aa residue 87/329 (Technical Appendix Figure 3).

To determine the prevalence of the *ccpA* (T87I) variant in CC30, we sequenced *ccpA* in an additional 34 *tst*-positive CC30 MRSA and 19 *tst*-positive CC30 MSSA isolates (Technical Appendix Table 1). Including genome-sequenced isolates, 33/39 *tst*-positive CC30 MRSA isolates had *ccpA* (T87I), compared with 0/23 *tst*-positive CC30 MSSA isolates, confirming an association of *ccpA* (T87I) with CC30 MRSA strains. Furthermore, *ccpA* (T87I) was strongly negatively associated with production of TSST-1 in *tst*-positive CC30 *S. aureus*: 26/33 *ccpA* (T87I) isolates did not produce TSST-1, compared with only 1/23 wild-type *ccpA* isolates (p<0.0001 by Fisher exact test).

We conducted SCC*mec* typing on a subset of *tst*-positive CC30 MRSA strains (n = 15; Technical Appendix Table 1). Results demonstrated an association of *ccpA* (T87I) with SCC*mec*II; 7/11 SCC*mec*II isolates had *ccpA* (T87I), compared with 0/4 SCC*mec*IV isolates (p = 0.03 by χ^2^ test). This finding highlights the possibility that reduced TSST-1 production might be attributable to either SCC*mec*II or *ccpA* (T87I).

## Discussion

We provide a substantive national clinical and microbiological overview of staphylococcal TSS cases in the United Kingdom. TSS incidence was 0.07/100,000 population, nmTSS cases now outnumber mTSS cases, and nmTSS affects younger persons. The *tst*-positive CC30 *S. aureus* lineage was linked strongly with TSS and almost all mTSS cases. CC30 MSSA is a prevalent lineage in the United Kingdom (*16*), so ongoing surveillance and clinical vigilance for TSS are important. 

Our findings may underestimate TSS incidence because notification of TSS is voluntary in the United Kingdom and we included only microbiologically confirmed cases. These factors increase diagnostic confidence, but TSS is a syndromic condition not requiring bacteriological confirmation. Overall TSS incidence was low but similar to rates in the United States (*2*); improvements in care may account for low overall incidence of TSS, because patients may not fulfill all of the criteria required by the case definition of TSS. The overall TSS incidence in children contrasts with findings from a British Pediatric Surveillance Unit study in which a higher incidence of combined streptococcal and staphylococcal TSS cases was reported (*30*).

The number of cases of mTSS fell from 2009 to 2012, such that nmTSS cases are now more common than mTSS cases, mirroring US trends (*31*). Patients in our study were younger than in US cohorts (*31**,**32*), and nmTSS patients were younger than those with mTSS. Most nmTSS cases occurred in children, with burns and SSTIs as the cause in 51.8% (29/56) of these cases. An association between nmTSS and increased mortality rate has been reported, although a high incidence of bacteremia may have affected the findings of that study (*33*). It is possible that we did not ascertain all cases of TSS, although we found no difference in reported deaths between mTSS and nmTSS cases or associations with age; the overall death rate was 5%.

The association of TSS, and particularly mTSS, with a single lineage corresponding to CC30 *S. aureus* has been described in diverse geographic localities (*14**,**15*). The *tst*-positive CC30 MSSA clone has recently been named epidemic MSSA-ST30 because it is responsible for a substantial amount of *S. aureus* disease and is a precursor to the HA-MRSA clone, EMRSA-16, which has been responsible for major national UK MRSA outbreaks (*29*).

The *tst* gene was the predominant superantigen gene among TSS isolates, excluding *seg* and *sei*, which were also previously implicated in TSS (*34*). The superantigens *seg* and *sei* are carried on the *egc*, which is widespread in *S. aureus* (*5*), and are unlikely to have any specific association with TSS. We linked *tst* to mTSS and CC30. Several groups have demonstrated similar associations of staphylococcal superantigen genes with specific lineages (*35**,**36*), due to clonal associations, superantigen arrangements, and transmission via mobile genetic elements, although other firm associations linking lineage, superantigen gene carriage, infection type, and disease presentations have not been made. A recent study of atopic dermatitis that examined the relationship of ethnicity and staphylococcal virulence factors found a lack of *tst*-positive *S. aureus* atopic dermatitis in African American persons that was consistent with an absence of *tst*-positive *S. aureus* mTSS among this group, suggesting differences in disease presentation among disparate ethnic groups (*37*) based on host characteristics. The ethnicity of the patients with TSS referred to PHE in this study was not recorded, and such bacterial genetic associations with disease could not be made but may merit consideration in future studies.

Among MSSA isolates, resistance rates to key antimicrobial drugs were similar to reported UK MSSA bacteremia isolates (*38*). Notably, teicoplanin resistance was detected, although rarely. This finding circumvents any need to change current recommendations for antimicrobial drugs for TSS that include a bactericidal cell wall inhibitor (e.g., β-lactamase–resistant antistaphylococcal) and protein-synthesis inhibitor (e.g., clindamycin) along with intravenous immunoglobulin for severe cases unresponsive to first-line therapy and source control (*39*). No vaccines are available to prevent TSS, although a recombinant TSST-1 variant vaccine has shown promise in a recent human clinical trial and was found to be safe and immunogenic (*40*).

The MRSA-TSS rate in this study was lower than rates in the United States (*32*), perhaps reflecting the low UK community-associated MRSA prevalence (*41*). All MRSA cases were nonmenstrual and mostly associated with recognized healthcare-associated MRSA clones, although we did not record the mode of acquisition. Only 1 CC30 MRSA (EMRSA-16) isolate caused TSS, even though CC30 is the main TSS-associated lineage; this finding mirrors the national decline in UK EMRSA-16 over time (*42*).

Isolates of *tst*-positive CC30 MSSA were more likely to produce TSST-1 in vitro and secreted almost 3 times more TSST-1 than did *tst*-positive CC30 MRSA isolates, which translated into a functional difference in superantigenic activity. We do not know whether such a difference would extend to the in vivo setting. Our study of TSST-1 production was limited by availability of clinical *tst*+ CC30 strains; clinical TSS CC30 MSSA strains were therefore compared with clinical non-TSS CC30 MRSA strains and not to clinical TSS MRSA strains. Thus, more MSSA than MRSA strains were from the genital tract or from burns, potentially confounding phenotypic differences observed. Defining the precise comparator group for TSS CC30 MSSA isolates is challenging because of lack of TSS CC30 MRSA isolates and suitable non-TSS strains referred to PHE.

Bacterial acquisition of antimicrobial drug resistance elements can be associated with a fitness cost. In the United Kingdom, most CC30 HA-MRSA strains carry SCC*mec*II (EMRSA-16; ST36-SCC*mec*II) that may reduce cytolytic toxin production and, in association with *fudoh* gene carriage by this element, reduce hemolytic activity and virulence (*43**,**44*). Our findings suggest an association between SCC*mec*II and reduced TSST-1 production that might be linked to a SNP in a regulatory gene, *ccpA*. The resulting mutation in CcpA occurs adjacent to a co-repressor binding site in the transcriptional regulation region (Technical Appendix Figure 3) that could influence *tst* promoter binding and affect TSST-1 secretion. Such SNPs in virulence regulators may have had a role in shaping the healthcare-associated phenotype of EMRSA-16 (*20*). New tools that allow manipulation of previously nontransformable lineages such as CC30 will facilitate investigating such genetic mechanisms in *S. aureus* (*45*).

Our study shows that the ability to produce TSST-1 varies widely within the *tst*-positive CC30 lineage and impaired expression is associated with the presence of SCC*mec*II and *ccpA* (T87I), underlining the potential for genomic approaches to contribute to greater understanding of patterns of clinical disease. Given the prevalence of *tst*-positive CC30 MSSA causing TSS and its role as a dominant UK lineage of *S. aureus*, active surveillance of this lineage is required. Clarification of the particular modes of transmission, acquisition, and pathogenesis of this lineage may identify susceptible persons, such as younger persons with burns and SSTIs, who might benefit from interventions such as vaccination with recombinant TSST-1 or *S. aureus* screening and decolonization in the future to prevent the occurrence of this life-threatening syndrome.

Technical Appendix Additional details on clinical and molecular epidemiology of staphylococcal toxic shock syndrome in the United Kingdom.
