# Supplementary material for: Clinical and Molecular Epidemiology of Staphylococcal Toxic Shock Syndrome in the United Kingdom
Source: Emerg Infect Dis. 2018 Feb;24(2):258–66. doi: 10.3201/eid2402.170606 (PMC5782905; doi:10.3201/eid2402.170606)
Supplement: Technical Appendix — Additional details on clinical and molecular epidemiology of staphylococcal toxic shock syndrome in the United Kingdom. [file 17-0606-Techapp-s1.pdf]

Article DOI: https://doi.org/10.3201/eid2402.170606

Clinical and Molecular Epidemiology of Staphylococcal Toxic Shock Syndrome in the United Kingdom

Technical Appendix

Methods

Bacterial Culture

We cultured bacterial isolates in 5 mL of brain heart infusion (BHI) for Western blotting or Roswell Park Memorial Institute (RPMI) media supplemented with 10% fetal calf serum (FCS) for proliferation assays, at 37°C with agitation. We removed bacterial cells from supernatants by centrifugation and 0.2µM filtration.

Analysis of TSST-1 by Western Blot

tst+ CC30 methicillin-sensitive *S. aureus* (MSSA) TSS-isolates (n = 81) and randomly selected *tst*+ CC30 methicillin-resistant *S. aureus* (MRSA) isolates (n = 39) (Technical Appendix Table 1) were cultured to stationary phase in BHI and supernatants prepared as above and then concentrated x10 using a 10 kDa spin column (Amicon, Merck Millipore, Nottingham, UK). Standard concentrations of purified TSST-1 (Toxin Technology, Sarasota, FL, USA) and bacterial supernatants were diluted 2:1 with NuPAGE LDS sample buffer (4x) (Life Technologies, Hemel Hempstead, UK) and 100 mM dithiothreitol then heated to 70°C for 10 minutes. A 15 µL sample was loaded onto 10% NuPAGE novex bis-tris gels. After electrophoresis, we transferred proteins to a PVDF membrane (Amersham Hybond-LFP, GE Healthcare Life Sciences, Amersham, UK) then blocked with 5% milk (Sigma, Dorset, UK) with 0.05% Tween-20 (Sigma, Dorset, UK). We incubated the samples overnight at 4°C with rabbit anti-TSST-1 polyclonal primary antibody (Abcam, Cambridge, UK) diluted 1:10,000; washed the blots and incubated them with anti-rabbit-HRP conjugated secondary antibody (Life Technologies) diluted 1:50,000; then developed them using Amersham ECL Prime Western Blotting Detection Reagent (GE Healthcare Life Sciences, Amersham, UK). We determined TSST-1

concentration in supernatants by comparing them with a TSST-1 standard curve by densitometry (LabWorks, UVP, Upland, CA, USA).

Referral Form

H2

PHE Microbiology request form

Public Health England

Healthcare Pathogens

Characterisation and Resistance (single isolate)

Bacteriology Reference Department (AMRHAI)
61 Colindale Avenue, London NW9 5HT

Phone: +44 (0)20 8327 7887
AMRHAI@phe.gov.uk
www.gov.uk/phe

PHE Colindale
Bacteriology
DX 6530002
Colindale NW

Please write clearly in dark ink

SENDER'S INFORMATION

Sender's name and address

Postcode

Report to be sent FAO

Contact Phone Ext

Purchase order number

Project code

PHE outbreak/investigation

ILog number

PATIENT/SOURCE INFORMATION

☐ Human ☐ Animal* ☐ Food* ☐ Water* ☐ Environment* ☐ Other* — *Please specify

☐ InPatient ☐ Outpatient ☐ GP Patient ☐ Other* — *Please specify

NHS number

Surname

Forename

Sex ☐ male ☐ female

Date of birth D D M M Y Y Y Y Age

Patient's postcode

Patient's HPT

Hospital number

Hospital name *(if different from sender's name)*

Ward/ clinic name

Ward type

☐ Medico-legal case

SAMPLE INFORMATION

Your reference

Isolation site

☐ Blood ☐ Nose ☐ Wound

☐ Environment ☐ Skin ☐ Urine

☐ Faeces ☐ Sputum

☐ Other *(please specify)*

Date of collection D D M M Y Y Time

Date sent to PHE D D M M Y Y

Priority status

Do you suspect that the isolate you are referring could be hazard group 3 ? ☐ Yes ☐ No

Please provide preliminary ID and laboratory results

Presumptive identification

☐ *S. aureus* MRSA ☐ *B. cepacia* complex ☐ *Klebsiella*

☐ *S. aureus* MSSA ☐ *Enterobacter* ☐ *P. aeruginosa*

☐ Coag Neg Staph ☐ *Enterococcus* ☐ *Serratia*

☐ *Acinetobacter* ☐ *E. coli* ☐ *S. maltophilia*

☐ *Other *(please specify)*

Hazard group 3 isolates (please telephone 020 8327 7233 to arrange)

☐ *Brucella spp* ☐ *B. pseudomallei* ☐ Other HG 3*

TESTS REQUESTED

☐ Typing *(please specify)*

☐ PVL toxin gene detection only *(S. aureus only)* ☐ Identification

☐ Extended toxin gene detection *(S. aureus only)* ☐ Genomovar determination *(B. cepacia - complex only)*

☐ MIC evaluation *(Specify reason below)*

☐ ESBL detection ☐ *mecA* PCR

☐ Carbapenem resistance ☐ *mupA* PCR

☐ Acquired AmpC ☐ Linezolid resistance

SENDER'S LABORATORY RESULTS

API profile no Gram stain

Oxidase +/- Catalase +/- Growth requirement

CLINICAL/EPIDEMIOLOGICAL INFORMATION

Clinical details

☐ Abscess ☐ Pyrexia/Fever

☐ Bacteraemia ☐ Septic shock

☐ Chest infection ☐ Septicaemia

☐ Cystic fibrosis ☐ Scalded skin syndrome

☐ Endocarditis ☐ Sudden infant death syndrome

☐ Fatal ☐ Toxic shock syndrome

☐ Pneumonia

☐ Other *(please specify)*

Reasons for request

☐ Confirmation of results ☐ Pseudobacteraemia

☐ Unusual resistance *(specify)* ☐ Sporadic

☐ Therapeutic guidance ☐ Suspected hospital acquired

☐ Continuing investigation ☐ Suspected community acquired

☐ Increasing numbers ☐ Suspected community MRSA

☐ Inter-hospital transfer

☐ Other *(please specify)*

Foreign Travel? ☐ Yes ☐ No Country

All requests are subject to PHE standard terms and conditions.

Version effective from Apr - 2014 BRDW0140.01

Technical Appendix Table 1. *Staphylococcus aureus* strains that caused toxic shock syndrome in the United Kingdom, 2008–2012*

Strain	Site of infection†	*mecA*	SCC*mec*	MLST-CC	*ccpA* (T87I)	Superantigen genes‡	TSST-1 ng/mL§
MSSA							
¶/#HSS354	mTSS	–	–	30	–	*seg, seh, sei, tst*	49.0
¶/#HSS355	mTSS	–	–	30	–	*sec, tst*	39.2
#HSS356	mTSS	–	–	30	–	*sea, seg, sei, tst*	112.5
¶/#HSS357	mTSS	–	–	30	–	*seg, sei, tst*	187.3
¶/#HSS358	mTSS	–	–	30	–	*seg, sei, tst*	154.5
#HSS359	Burn	–	–	30	–	*seg, sei, tst*	57.5
#HSS394	Abscess	–	–	30	ND	*sea, seg, sei, tst, pvl*	87.0
#HSS395	mTSS	–	–	30	–	*sea, seg, sei, tst*	124.8
#HSS397	Burn	–	–	30	–	*sea, seg, sei, tst*	73.3
#HSS398	Burn	–	–	30	ND	*seg, sei, tst*	38.8
#HSS405	Abscess	–	–	30	ND	*sea, seg, sei, tst*	57.1
#HSS409	mTSS	–	–	30	ND	*seg, sei, tst*	32.8
#HSS412	mTSS	–	–	30	–	*seg, sei, tst*	46.3
#HSS413	mTSS	–	–	30	ND	*seg, sei, tst*	40.1
#HSS414	mTSS	–	–	30	ND	*seg, seh, sei, tst*	42.3
#HSS416	mTSS	–	–	30	ND	*sea, seg, seh, sei, tst*	106.7
#HSS417	Skin	–	–	30	–	*seg, sei, tst*	25.8
#HSS419	mTSS	–	–	30	ND	*seg, sei, tst*	<25.0
#HSS422	mTSS	–	–	30	ND	*seg, sei, tst*	83.0
#HSS423	URT	–	–	30	–	*sea, seg, seh, sei, tst*	126.9
#HSS425	mTSS	–	–	30	ND	*sec, tst*	196.4
#HSS426	mTSS	–	–	30	ND	*sea, seg, sei, tst*	182.9
#HSS427	Skin	–	–	30	ND	*seg, sei, tst*	96.9
#HSS428	mTSS	–	–	30	ND	*sea, sec, seg, sei, tst*	57.0
#HSS429	mTSS	–	–	30	–	*seg, seh, sei, tst*	48.3
#HSS430	mTSS	–	–	30	ND	*seg, seh, sei, tst*	<25.0
#HSS431	mTSS	–	–	30	ND	*sea, seg, sei, tst*	55.2
#HSS432	mTSS	–	–	30	ND	*seg, seh, sei, tst*	120.0
#HSS434	Skin	–	–	30	ND	*seg, seh, sei, tst*	<25.0
#HSS435	mTSS	–	–	30	ND	*sea, seg, sei, tst*	54.9
#HSS436	mTSS	–	–	30	ND	*sea, seg, sei, tst*	125.2
#HSS437	Bacteremia	–	–	30	ND	*sea, seg, sei, tst*	161.4
#HSS438	mTSS	–	–	30	ND	*sea, seg, seh, sei, tst*	173.8
#HSS439	mTSS	–	–	30	ND	*sea, seg, sei, tst*	186.3
#HSS440	Burn	–	–	30	ND	*sea, seg, sei, tst*	129.9
#HSS441	mTSS	–	–	30	ND	*sea, seg, sei, tst*	61.8
#HSS443	mTSS	–	–	30	ND	*seg, seh, sei, tst*	108.2
#HSS445	UK	–	–	30	ND	*seg, seh, sei, tst*	105.7
#HSS446	LRT	–	–	30	ND	*sea, seg, sei, tst*	54.6
#HSS449	mTSS	–	–	30	–	*sea, seg, sei, tst*	134.3
#HSS451	mTSS	–	–	30	ND	*seg, sei, tst*	57.4
#HSS454	mTSS	–	–	30	ND	*sea, seg, sei, tst*	49.1
#HSS456	mTSS	–	–	30	ND	*seg, sei, tst*	51.9
#HSS457	mTSS	–	–	30	ND	*seg, sei, tst*	78.0
#HSS459	Skin	–	–	30	ND	*sea, seg, sei, tst*	54.2
#HSS463	mTSS	–	–	30	–	*seg, seh, sei, tst*	57.9
#HSS468	UK	–	–	30	ND	*sec, seg, sei, tst, pvl*	26.8
#HSS469	Bacteremia	–	–	30	–	*sea, seg, sei, tst*	133.6
#HSS470	mTSS	–	–	30	ND	*sea, seg, sei, tst*	96.0
#HSS473	Skin	–	–	30	–	*seg, seh, sei, tst*	92.4
#HSS474	mTSS	–	–	30	–	*sea, seg, sei, tst*	<25.0
#HSS475	mTSS	–	–	30	ND	*sec, seg, sei, tst*	94.4
#HSS476	mTSS	–	–	30	ND	*sec, seg, sei, tst*	108.3
#HSS478	mTSS	–	–	30	ND	*seg, sei, tst*	66.3
#HSS479	Skin	–	–	30	ND	*seg, sei, tst*	204.6
#HSS481	Skin	–	–	30	ND	*sea, seg, sei, tst*	40.0
#HSS485	mTSS	–	–	30	ND	*sea, seg, sei, tst*	68.7
#HSS490	UK	–	–	30	ND	*sea, seg, sei, tst*	174.8
#HSS492	Burn	–	–	30	ND	*seg, sei, tst*	82.9
#HSS493	Eye	–	–	30	ND	*sea, seg, sei, tst*	65.8
#HSS494	Bacteremia	–	–	30	ND	*sea, seg, sei, tst*	116.9
#HSS496	mTSS	–	–	30	ND	*sea, seg, sei, tst*	44.7
#HSS497	Bacteremia	–	–	30	ND	*seg, seh, sei, tst*	90.8
#HSS499	Abscess	–	–	30	ND	*sea, seg, sei, tst*	133.4
#HSS501	Skin	–	–	30	–	*seg, seh, sei, tst*	122.8
#HSS502	mTSS	–	–	30	ND	*seg, sei, tst*	98.0
#HSS503	Skin	–	–	30	ND	*sea, seg, sei, tst*	111.6
#HSS504	mTSS	–	–	30	ND	*sea, seg, sei, tst*	60.0
#HSS512	mTSS	–	–	30	–	*sea, seg, seh, sei, tst*	37.6
#HSS513	Burn	–	–	30	–	*sea, seg, sei, tst*	66.9

Strain	Site of infection†	*mecA*	SCC*mec*	MLST-CC	*ccpA* (T87I)	Superantigen genes‡	TSST-1 ng/mL§
#HSS514	mTSS	–	–	30	ND	*sea, seg, seh, sei, tst*	74.9
#HSS517	mTSS	–	–	30	ND	*sea, seg, sei, tst*	30.4
#HSS518	mTSS	–	–	30	ND	*sea, seg, sei, tst*	70.2
#HSS520	mTSS	–	–	30	ND	*sea, seg, sei, tst*	79.8
#HSS521	mTSS	–	–	30	–	*sea, seg, sei, tst*	61.8
#HSS522	Skin	–	–	30	ND	*sea, seg, sei, tst*	75.8
#HSS525	Skin	–	–	30	ND	*sea, seg, sei, tst*	168.4
#HSS527	Skin	–	–	30	ND	*sea, seg, sei, tst*	134.9
#HSS530	Abscess	–	–	30	ND	*seg, sei, tst*	45.1
#HSS533	UK	–	–	30	–	*sea, seg, sei, tst*	114.6
#HSS535	mTSS	–	–	30	–	*sec, tst*	191.6
MRSA							
¶HSS360	Urine	+	ND	30	+	*sea, seg, sei, tst*	<25.0
HSS361	Bone and joint	+	ND	30	+	*sea, seg, sei, tst*	<25.0
¶/#HSS362	Abscess	+	II	30	+	*sea, seg, sei, tst*	<25.0
¶HSS363	Bacteremia	+	ND	30	+	*sea, seg, sei, tst*	<25.0
¶HSS364	Bacteremia	+	ND	30	+	*seg, sei, tst*	<25.0
HSS377	UK	+	IV	30	–	*seg, sei, tst*	<25.0
HSS378	UK	+	IV	30	–	*seg, sei, tst*	<25.0
¶HSS379	UK	+	II	30	+	*seg, sei, tst*	<25.0
HSS537	Skin	+	ND	30	+	*sea, seg, sei, tst*	<25.0
HSS538	Skin	+	ND	30	+	*sea, seg, sei, tst*	<25.0
HSS539	Sputum	+	ND	30	+	*seg, sei, tst*	<25.0
HSS540	Bacteremia	+	ND	30	+	*sea, seg, tst*	<25.0
HSS542	Nose	+	ND	30	+	*sea, seg, sei, tst*	<25.0
HSS543	Nose	+	II	30	+	*sea, seg, sei, tst*	94.5
HSS544	Bacteremia	+	II	30	+	*sea, seg, sei, tst*	63.1
HSS545	UK	+	ND	30	+	*seg, sei, tst*	<25.0
HSS546	Nose	+	ND	30	+	*sea, seg, sei, tst*	<25.0
HSS548	Bacteremia	+	II	30	–	*sea, seg, sei, tst*	<25.0
HSS549	Sputum	+	ND	30	+	*sea, seg, sei, tst*	<25.0
HSS550	Skin	+	IV	30	–	*seg, sei, tst*	76.3
HSS551	Skin	+	ND	30	+	*sea, seg, sei, tst*	<25.0
HSS552	Nose	+	IV	30	–	*sea, seg, sei, tst*	97.5
HSS553	Eye	+	ND	30	+	*sea, seg, sei, tst*	25.8
HSS555	Skin	+	II	30	+	*sea, seg, sei, tst*	31.2
HSS556	Abscess	+	ND	30	+	*seg, sei, tst*	<25.0
HSS558	Throat	+	ND	30	+	*sea, seg, tst*	<25.0
HSS559	Skin	+	II	30	+	*sea, seg, sei, tst*	38.5
HSS560	UK	+	II	30	–	*sea, seg, sei, tst*	<25.0
HSS561	Bacteremia	+	ND	30	+	*sea, seg, sei, tst*	<25.0
HSS562	Throat	+	ND	30	+	*sea, seg, sei, tst*	<25.0
HSS563	Nose	+	II	30	+	*sea, seg, tst*	27.0
HSS564	Throat	+	ND	30	+	*sea, seg, sei, tst*	<25.0
HSS565	Skin	+	ND	30	+	*sea, seg, sei, tst*	<25.0
HSS566	Sputum	+	ND	30	+	*sea, seg, sei, tst*	<25.0
HSS570	Nose	+	II	30	+	*sea, seg, sei, tst*	<25.0
HSS571	Bacteremia	+	II	30	+	*sea, seg, sei, tst*	26.7
HSS573	Nose	+	ND	30	+	*sea, seg, sei, tst*	<25.0
HSS574	Nose	+	ND	30	+	*sea, seg, sei, tst*	<25.0
HSS575	Nose	+	ND	30	+	*sea, seg, sei, tst*	<25.0

*MLST-CC, multilocus sequence type-clonal complex (inferred from *spa* typing data); MRSA, methicillin-resistant *S.aureus*; MSSA, methicillin-sensitive *S.aureus*; mTSS, menstrual toxic shock syndrome; ND, not done; SCC*mec*, staphylococcal cassette chromosome *mec* element; UK, unknown. Boldface indicates MSSA or MRSA isolates.
†MSSA isolates were all from TSS cases. Site of infection specified only for nonmenstrual TSS isolates.
‡Toxin gene profile was determined by multiplex PCR that detected *sea-see, seg-sej, tst* and *pvl.*
§TSST-1 production measured by immunoblot, limit of detection 25ng/mL.
¶Strains that were subject to whole genome sequencing; data deposited in the GenBank short read archive (accession no. SRP082305).
#CC30 strains tested for antimicrobial susceptibility.

Technical Appendix Table 2. Clinical characteristics of fatal cases of toxic shock syndrome in England, Wales, and Northern Ireland, 2008–2012*

Characteristics	Fatal cases n = 9	Nonfatal cases n = 171	p-value
Median age, y (IQR)	36 (6–51)	19 (9–38)	0.39†
Sex, no. (%)			
Female	7 (77.8)	121 (70.8)	1.00‡
Male	2 (22.2)	49 (28.6)	1.00‡
Unknown	0	1 (0.6)	
Type of TSS, no. (%)§			
Menstrual	4 (44.4)	102 (56.7)	
Nonmenstrual	5 (55.6)	66 (36.7)	0.74‡

*IQR, interquartile range; TSS, toxic shock syndrome.
†Mann-Whitney U test comparing fatal and nonfatal cases.
‡Fisher exact test comparing fatal and nonfatal cases.
§3 case isolates not assigned as mTSS or nmTSS due to lack of clinical data.

Technical Appendix Table 3. The clonal complexes and associated *spa*-types of isolates causing menstrual and nonmenstrual toxic shock syndrome in England, Wales, and Northern Ireland, 2008–2012*

	Menstrual (n = 70)		Nonmenstrual (n = 107)		
MLST-CC	***spa* types**	**no. (%)**	***spa* types**	**no. (%)**	p-value†
Unknown	NA	2 (2.9)	NA	12 (11.2)	
1	NA	0	t127, t922	5 (4.7)	
5	t002, t6614	3 (4.3)	t002, t045, t548, t688, t7348	9 (8.4)	0.37
6	NA	0	t304	1 (0.9)	
8	t197, t1188, t12650	3 (4.3)	t008, t104, t723	5 (4.7)	
12	t160	1 (1.4)	t156, t160	3 (2.8)	
15	t084	1 (1.4)	t084, t085, t091, t774	6 (5.6)	
22	t223	2 (2.9)	t005, t020, t022, st032, t223, t379, t9606	8 (7.5)	0.32
25	t078	2 (2.9)	t167, t937	2 (1.9)	
30	t012, t018, t019, t021, t089, t136, t166, t338, t399, t440, t582, t862, t870, t942, t2018, t2387, t2868, t3072, t3368, t3687, t4242, t6359, t12601, t12649	51 (72.9)	t012, t018, t019, t021, t122, t166, t275, t338, t414, t1298, t1675, t2895, t3233, t3800, t4077, t5753, t6364, t6424, t11323	39 (36.4)	**<0.0001**
45	t026, t230	2 (2.9)	t015, t065, t230, t383, t465, t583, t2642, t2887	10 (9.3)	0.12
59	t7467	1 (1.4)	t216, t437, t471	4 (3.7)	
97	t359	1 (1.4)	NA	0	
121	NA	0	t171, t314	2 (1.9)	
182	NA	0	t364	1 (0.9)	
398	t571	1 (1.4)	NA	0	

*MLST-CC, Multilocus sequence type-clonal complex (inferred from *spa* typing data); NA, not applicable. Boldface indicates a statistically significant result. 3 case isolates not assigned as mTSS or nmTSS due to lack of clinical data
†Fisher exact test comparing MLST-CC between menstrual and nonmenstrual toxic shock syndrome case-patients in MLST-CC groups with ≥10 isolates.

Technical Appendix Table 4. The contribution of methicillin-resistant *S. aureus* isolates to toxic shock syndrome cases, 2008–2012*

Attribute	MRSA n = 7	MSSA n = 173	p-value
Clinical characteristics, no. (%)			
Menstrual	0 (0)	70 (40.5)	
Nonmenstrual	7 (100)	100 (57.8)	**0.04**†
Median age, y (IQR)	34 (2.3–64.3)	19 (10–38.5)	0.39‡
Sex, no. (%)§			
Female	2 (28.6)	126 (72.8)	
Male	5 (71.4)	46 (26.6)	**0.02†**
Molecular characteristics, no. (%)			
MLST-CC/SCC*mec*			
6/II	1 (14.3)	16 (8.7)	1.00
22/IV	5 (71.4)	5 (2.9)	**<0.0001†**
30/II	1 (14.3)	91 (52.6)	0.06†
Superantigens, no. (%)			
sea and *tst*	1 (14.3)	51 (29.5)	0.68†
sec	4 (57.1)	10 (5.8)	**0.0007†**
tst	2 (28.6)	101 (58.4)	0.14†

*MLST-CC, Multilocus sequence type-clonal complex (inferred from *spa* typing data); MRSA, Methicillin resistant *S. aureus*; MSSA, Methicillin sensitive *S. aureus*; SCC*mec*, Staphylococcal Cassette Chromosome *mec* element. Boldface indicates a statistically significant result. 3 case isolates not assigned as mTSS or nmTSS due to lack of clinical data.
†Fisher exact test.
‡Mann-Whitney U test.
§Sex of 1 nmTSS case-patient is unknown

Technical Appendix Table 5. Superantigen gene frequency of *sea-sed* in each *S. aureus* clonal complex causing toxic shock syndrome, 2008–2012*

MLST-CC	No. (%) isolates n = 180	Mean no. superantigen genes/CC	Superantigen, no. (%) positive isolates *sea*	*seb*	*sec*	*sed*
1	5 (2.8)	2.6	4 (80.0)	1 (20.0)	0	0
5	12 (6.7)	3.1	0	0	2 (16.7)	4 (33.3)
6	1 (0.6)	0.0	0	0	0	0
8	8 (4.4)	1.4	4 (50.0)	1 (12.5)	0	1 (12.5)
12	4 (2.2)	1.8	1 (25.0)	2 (50.0)	1 (25.0)	0
15	7 (3.9)	0.0	0	0	0	0
22	10 (5.6)	2.9	0	0	4 (40.0)	0
25	4 (2.2)	2.0	0	2 (50.0)	0	0
30	92 (51.1)	3.7	**48 (52.2)†**	1 (1.1)	8 (8.7)	0
45	12 (6.7)	2.8	0	1 (8.3)	**7 (58.3)‡**	1 (8.3)
59	5 (2.8)	1.2	1 (20.0)	2 (40.0)	0	0
97	1 (0.6)	0.0	0	0	0	0
121	2 (1.1)	2.0	0	0	0	0
182	1 (0.6)	3.0	0	0	0	0
398	1 (0.6)	0.0	0	0	0	0
Other	15 (8.3)	2.7	6 (40.0)	3 (20.0)	1 (6.6)	4 (26.7)

*MLST-CC, Multilocus sequence type-clonal complex (inferred from *spa* typing data). The superantigen gene *see* was not detected in any isolate. Boldface indicates a statistically significant result.Percentages may not total 100 due to rounding.
†p<0.0001 by Fisher exact test comparing the percentage carriage of a given superantigen gene by an individual CC to percentage carriage of the same superantigen gene by all other CCs combined.
‡p< 0.001 by Fisher exact test comparing the percentage carriage of a given superantigen gene by an individual CC to percentage carriage of the same superantigen gene by all other CCs combined.

Technical Appendix Table 6. Superantigen gene distribution of *seg-tst* in each *S. aureus* clonal complex causing toxic shock syndrome, 2008–2012*

MLST-CC	No. (%) isolates n = 180	Superantigen, no. (%) positive isolates *seg*	*seh*	*sei*	*sej*	*tst*
1	5 (2.8)	1 (20.0)	5 (100.0)	1 (20.0)	0	1 (20.0)
5	12 (6.7)	12 (100.0)	0	12 (100.0)	4 (33.3)	3 (25.0)
6	1 (0.6)	0	0	0	0	0
8	8 (4.4)	0	0	0	3 (37.5)	2 (25.0)
12	4 (2.2)	1 (25.0)	0	1 (25.0)	0	1 (25.0)
15	7 (3.9)	0	0	0	0	0
22	10 (5.6)	10 (100.0)	0	10 (100.0)	0	5 (50.0)
25	4 (2.2)	3 (75.0)	0	3 (75.0)	0	0
30	92 (51.1)	**87 (94.6)†**	**18 (19.6)‡**	**87 (94.6)†**	0	**89 (96.7)†**
45	12 (6.7)	11 (91.7)	0	12 (100.0)	1 (8.3)	1 (8.3)
59	5 (2.8)	1 (20.0)	0	1 (20.0)	0	1 (20.0)
97	1 (0.6)	0	0	0	0	0
121	2 (1.1)	2 (100.0)	0	2 (100.0)	0	0
182	1 (0.6)	1 (100.0)	1 (100.0)	1 (100.0)	0	0
398	1 (0.6)	0	0	0	0	0
Other	15 (8.3)	9 (60.0)	1 (6.6)	9 (60.0)	4 (26.7)	3 (20.0)

*MLST-CC, Multilocus sequence type-clonal complex (inferred from *spa* typing data). The superantigen gene *see* was not detected in any isolate. Boldface indicates a statistically significant result.
†p < 0.0001 by Fisher exact test comparing the percentage carriage of a given superantigen gene by an individual CC to percentage carriage of the same superantigen gene by all other CCs combined.
‡p < 0.05 by Fisher exact test comparing the percentage carriage of a given superantigen gene by an individual CC to percentage carriage of the same superantigen gene by all other CCs combined.

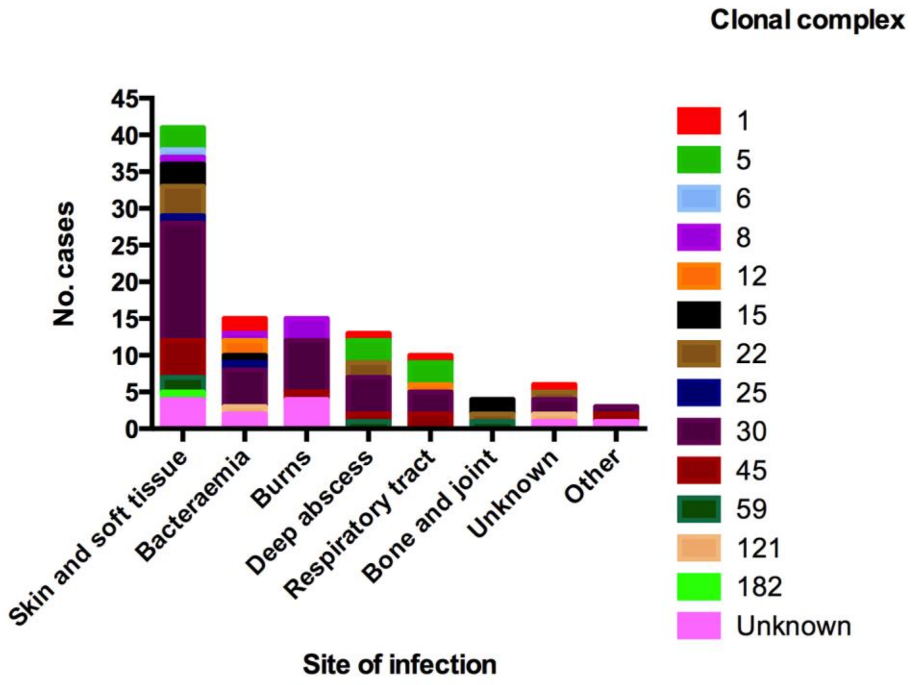

Technical Appendix Figure 1. Foci of infection and clonal complexes of *S. aureus* isolates causing nonmenstrual toxic shock syndrome in England, Wales, and Northern Ireland, 2008–2012. The figure shows numbers of isolates from each clonal complex causing 107 nonmenstrual TSS cases by focus of infection. "Other" includes one of each of eye, gastrointestinal, and genitourinary tracts. "Clonal complex: Unknown" refers to isolates that failed to grow on sub-culture.

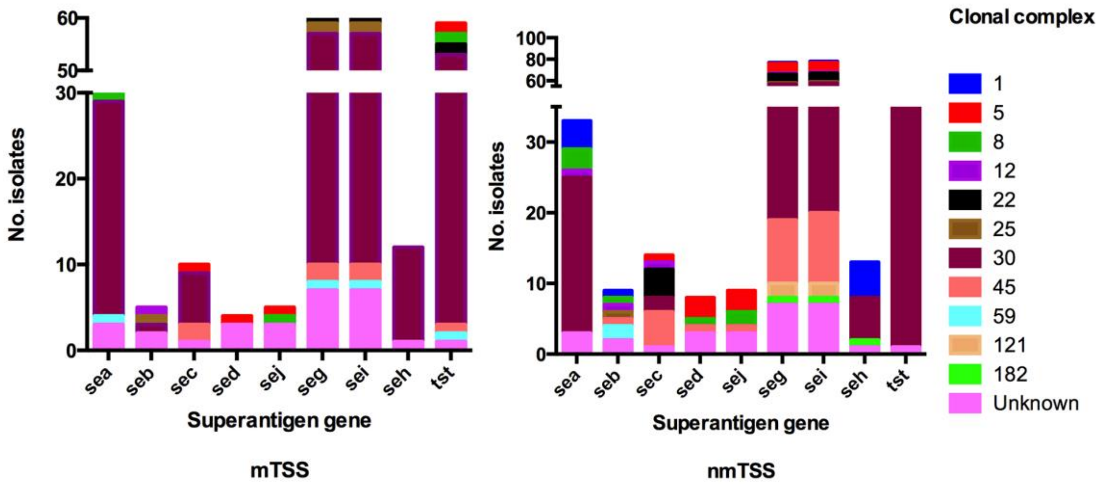

Technical Appendix Figure 2. Superantigen gene frequency and clonal complexes of *S. aureus* isolates causing toxic shock syndrome in England, Wales and Northern Ireland, 2008–2012. The figure shows number of isolates from each clonal complex carrying each superantigen gene in A) menstrual and B) nonmenstrual TSS cases; *see* was not detected in any isolate. mTSS, menstrual toxic shock syndrome; nmTSS, nonmenstrual toxic shock syndrome "Clonal complex: Unknown" refers to isolates that failed to grow on subculture.

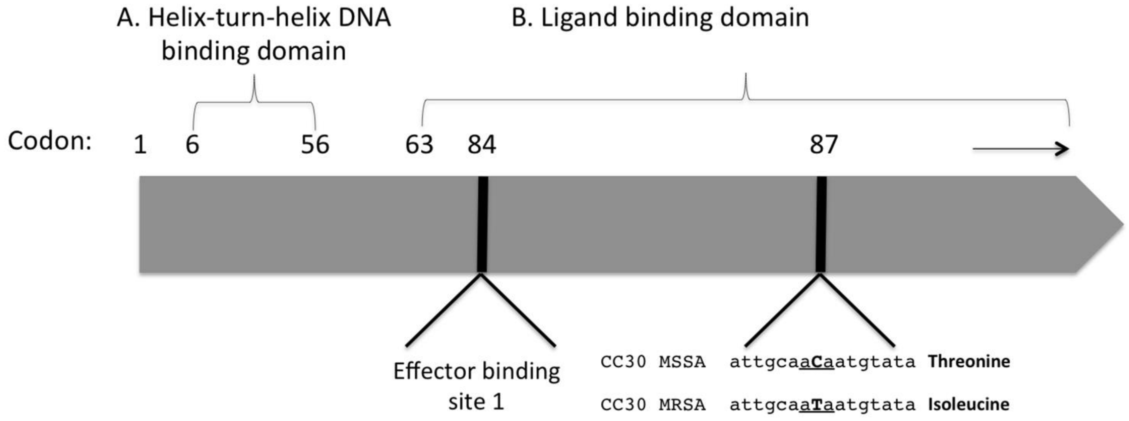

Technical Appendix Figure 3. The amino acid sequence of CcpA in CC30 MSSA and CC30 MRSA. Region A represents the helix-turn-helix DNA binding domain of the LacI family of transcriptional regulators to which CcpA belongs. Region B represents the ligand binding domain, which is the major transcriptional regulator. The solid vertical bars represent potentially important residues. Effector binding site 1 at position 84 is one of 8 residues where the key co-repressor phosphoprotein (HPr) binds to CcpA; adjacent to this at position 87 is the amino acid change from Threonine in CC30 MSSA to Isoleucine in CC30 MRSA (T87I). This has been expanded to illustrate the change in the nucleotide sequence from C in CC30 MSSA to T in CC30 MRSA at base pair 257 from the transcriptional start site.
